# Supplementary material for: Marginal speed confinement resolves the conflict between correlation and control in collective behaviour
Source: Nat Commun. 2022 May 10;13:2315. doi: 10.1038/s41467-022-29883-4 (PMC9090766; doi:10.1038/s41467-022-29883-4)
Supplement: Supplementary file 1 — Supplementary information for Marginal speed confinement resolves the conflict between correlation and control in collective behaviour [file 41467_2022_29883_MOESM1_ESM.pdf]

Supplementary Information for
Marginal speed confinement resolves the conflict between correlation and control in collective behaviour

A. Cavagna, A. Culla, X. Feng, I. Giardina, T. S. Grigera, W. Kion-Crosby, S. Melillo, G. Pisegna, L. Postiglione and P. Villegas

Distribution of the mean speed: linear speed control

In this section we describe how to derive the approximate mean speed distribution of Eq. (6) in the main text. The starting point is the pseudo-Hamiltonian with the harmonic potential:

$$H(\{\boldsymbol{v}_i\}) = \frac{J}{2}\sum_{i,j} n_{ij}(\boldsymbol{v}_i - \boldsymbol{v}_j)^2 + g\sum_i (v_i - v_0)^2 \tag{S1}$$

where all the sums are from 1 to the number of particles in the system N. We are dealing with an active system, hence the matrix $n_{ij} = n_{ij}(t)$ depends on time. However, it has been shown in [1] that, due to the large polarization of real flocks, the relaxation time scale of $n_{ij}(t)$ is significantly larger than that of the velocities, so that a quasi-equilibrium approach to the problem is reasonable; from now on we will then consider a time-independent n_{ij}. The validity of this approach is retrospectively confirmed by the remarkable agreement between the predictions of the approximate equilibrium theory derived here below, and the results from self propelled particles simulations, as displayed in Fig.3b and Fig.3d of the main text. In the context of quasi-equilibrium, we can assume a Boltzmann-like distribution for the velocities

$$P(\{\boldsymbol{v}_i\}) = \frac{1}{Z}\exp\left(-\beta H(\{\boldsymbol{v}_i\})\right) , \tag{S2}$$

where $\beta = 1/T$ is the inverse temperature, and quantifies the degree of noise in the system. Our aim is now to marginalize (S2) to get a probability distribution for the mean speed (notice that, although the confining potential is harmonic, it is so in the *speed*, i.e. the modulus of the velocity, $|\mathbf{v}_i|$, which is *not* a linear function of $\mathbf{v}_i$; hence, the model is in fact not strictly linear). It is convenient to rewrite (S1) in terms of the individual speeds $v_i = |\boldsymbol{v}_i|$ and flight directions $\boldsymbol{\sigma}_i = \boldsymbol{v}_i/v_i$. In the very ordered phase, one can use the "spin-wave approximation" (SW) [2], as already done in previous analysis of starling flocks [3, 4]. When the polarization is large (enforced in our model by choosing $J \gg 1$), the flight direction of each individual is very close to the polarization vector. Hence:

$$\boldsymbol{v}_i = v_i\boldsymbol{\sigma}_i \qquad \text{with} \qquad |\boldsymbol{\sigma}_i| = 1 \tag{S3}$$

$$\boldsymbol{\sigma}_i \simeq \boldsymbol{n}\left(1 - \frac{\pi_i^2}{2}\right) + \boldsymbol{\pi}_i \tag{S4}$$

where $\boldsymbol{n}$ is the unit vector along the polarization vector $\boldsymbol{\Phi} = \frac{1}{N}\sum_i \boldsymbol{\sigma}_i$, and the $\boldsymbol{\pi}_i$ are the fluctuations orthogonal to $\boldsymbol{n}$. The constraint $\sum_i \boldsymbol{\pi}_i = 0$ holds by construction and, in the high ordered regime, $\pi_i^2 \ll 1$ for every i. The Hamiltonian (S1) then becomes, up to order π_i^2:

$$H(\{v_i\},\{\boldsymbol{\pi}_i\}) = J\sum_{i,j}\Lambda_{ij}v_iv_j + g\sum_i (v_i - v_0)^2 + J\sum_{i,j}\tilde{\Lambda}_{ij}(\{v_k\})\boldsymbol{\pi}_i\cdot\boldsymbol{\pi}_j , \tag{S5}$$

where we defined the matrices:

$$\Lambda_{ij} = -n_{ij} + \delta_{ij}\sum_k n_{ik} \qquad \text{(Discrete Laplacian)} \tag{S6}$$

$$\tilde{\Lambda}_{ij}(\{v_k\}) = -n_{ij}v_iv_j + \delta_{ij}\sum_k n_{ik}v_iv_k . \tag{S7}$$

In terms of the variables $\{v_i\}$ and $\{\boldsymbol{\pi}_i\}$, the probability density (S2) becomes,

$$P(\{v_i\},\{\boldsymbol{\pi}_i\}) = \frac{\delta\left(\sum_k \boldsymbol{\pi}_k\right)\prod_i v_i^{d-1} e^{-\beta H}}{\int \mathrm{D}v' \mathrm{D}\boldsymbol{\pi}' \delta\left(\sum_k \boldsymbol{\pi}'_k\right) e^{-\beta H}\prod_i v_i'^{d-1}} \tag{S8}$$

where $\mathrm{D}v' \equiv \prod_k \mathrm{d}v'_k$, $\mathrm{D}\boldsymbol{\pi}' \equiv \prod_k \mathrm{d}\boldsymbol{\pi}'_k$ and d is the dimension of the velocity vector. We now need to integrate out the fluctuations $\boldsymbol{\pi}_i$, to obtain the marginalized distribution of the individual speeds v_i. Let us define

$$\Omega(\{v_i\}) \equiv \prod_j v_j^{d-1} \int \mathrm{D}\boldsymbol{\pi} \exp\left[-\beta J \sum_{i,j} \tilde{\Lambda}_{ij}(\{v_k\}) \boldsymbol{\pi}_i \cdot \boldsymbol{\pi}_j\right] \delta\left(\sum_k \boldsymbol{\pi}_k\right) . \tag{S9}$$

The integral can be easily performed upon a change of integration variables from the $\{\boldsymbol{\pi}_i\}$ to the eigenvectors $\{\tilde{\boldsymbol{\pi}}_\alpha\}$ of the matrix $\tilde{\Lambda}$. Both Λ and $\tilde{\Lambda}$ inherit the translational invariance of the original Hamiltonian and have a constant eigenvector corresponding to a zero mode, since $\sum_j \Lambda_{ij} = \sum_j \tilde{\Lambda}_{ij} = 0$. The constraint on the $\{\boldsymbol{\pi}_i\}$ becomes a constraint on the zero mode, i.e. $\delta(\tilde{\boldsymbol{\pi}}_0)$, making the integral finite and leaving out only $d-1$ eigenvalues. We get

$$\Omega(\{v_i\}) = \left[\prod_j v_j^{d-1}\right]\left[\prod_{\alpha\neq 0} \tilde{\lambda}_\alpha(\{v_k\})\right]^{-\frac{d-1}{2}} \tag{S10}$$

where the $\{\tilde{\lambda}_\alpha\}$ are the eigenvalues of $\tilde{\Lambda}$ and depend on the $\{v_i\}$ in some complicated way. Since we are interested in the distribution of the mean speed $s = (1/N)\sum_i v_i$, we will now estimate the behaviour of Ω to leading order in s. Once again, it is convenient to make a change of variables, going from real space to the space of the eingenvectors $\{\hat{v}_a\}$ of the discrete Laplacian Λ. Each v_i can be decomposed into its $\hat{v}_a$ components using the formula $v_i = \sum_a w_i^{(a)} \hat{v}_a$, where $w_i^{(a)}$ is the change of basis matrix. As mentioned above, the zero-mode has constant coefficients $w_i^{(0)} = 1/\sqrt{N}$ and the zero-mode eigenvector is therefore proportional to the mean speed, i.e. it is exactly $\sqrt{N}s = \left(1/\sqrt{N}\right)\sum_i v_i$. This also implies that for each v_i we have,

$$v_i = s + \delta v_i = s + \sum_{a\neq 0} w_i^{(a)} \hat{v}_a \ . \tag{S11}$$

We can now express the function Ω, in terms of this new representation

$$\Omega \sim \frac{\prod_j v_j^{d-1}}{\left[\prod_{\alpha\neq 0} \tilde{\lambda}_\alpha(\{v_k\})\right]^{\frac{d-1}{2}}} = \frac{\prod_j \left[s + \sum_{a\neq 0} w_j^{(a)}\hat{v}_a\right]^{d-1}}{f\left(\left\{s + \sum_{a\neq 0} w_k^{(a)}\hat{v}_a\right\}\right)} = s^{d-1}\frac{\prod_j \left[1 + \sum_{a\neq 0} \frac{w_j^{(a)}\hat{v}_a}{s}\right]^{d-1}}{f\left(\left\{1 + \sum_{a\neq 0} \frac{w_k^{(a)}\hat{v}_a}{s}\right\}\right)} = s^{d-1} h\left(\left\{1 + \sum_{a\neq 0} \frac{w_k^{(a)}\hat{v}_a}{s}\right\}\right) . \tag{S12}$$

Here h is a generic rational function of its argument. The function f is a generic polynomial of order $(N-1)(d-1)$ in its argument (from dimensional analysis), hence it is safe to extract a $s^{(N-1)(d-1)}$, because s is present in the expansion of every v_k.

The term Ω describes the contribution to the measure coming from the integration of the directional fluctuations. Once we integrate the directional fluctuations, we have an Hamiltonian that only depends on the moduli $\{v_i\}$. Also in this case, we can express everything in terms of s and the non-zero modes $\{\hat{v}_a\}$ of Λ. Remembering that $\sum_i w_i^{(a)} w_i^{(b)} = \delta_{a,b}$, we get,

$$H = J\sum_{i,j} \Lambda_{ij} v_i v_j + g\sum_i (v_i - v_0)^2 = \sum_{a=1}^{N} (J\lambda_a + g)\hat{v}_a^2 + gN(s - v_0)^2 \tag{S13}$$

where, with a slight abuse of notation, we still indicate with H the marginalised Hamiltonian depending only on the speeds. After these manipulations we get the distribution,

$$P(\{s,\hat{v}_a\}) = \frac{\Omega(\{s,\hat{v}_a\})\ e^{-\beta H}}{\int \mathrm{d}s'\ \mathrm{D}\hat{v}'\ \Omega(\{s',\hat{v}'_b\})\ e^{-\beta H}} \ , \tag{S14}$$

with $a \neq 0$ and $\mathrm{D}\hat{v'} \equiv \prod_{b\neq 0} \mathrm{d}\hat{v'}_b$. We can now derive the distribution of the mean speed $s = \frac{1}{N}\sum_i v_i$ by marginalizing over all the non-zero modes $\hat{v}_a$. To this end, we note that since $\left|w_i^{(a)}\right| < 1$ for every i and a, we have $\hat{v}_a = \sum_i w_i^{(a)} v_i < \sum_i v_i = Ns$. The domain of the variables appearing in (S14) is therefore,

$$0 \le s < \infty \tag{S15}$$

$$-Ns \le \hat{v}_a \le Ns \qquad \text{for } a \neq 0 \ . \tag{S16}$$

We then get,

$$\begin{aligned} P(s) &= \frac{1}{Z_s} \exp\left[-N\beta g (s-v_0)^2\right] \int_{-Ns}^{Ns} \mathrm{D}\hat{v}\ \Omega(s, \{\hat{v}_a\})\ \exp\left[-\beta \sum_{a=1}^{N} (J\lambda_a + g)\,\hat{v}_a^2\right] \\ &= \frac{1}{Z_s} s^{d-1} \exp\left[-N\beta g (s-v_0)^2\right] \int_{-Ns}^{Ns} \mathrm{D}\hat{v}\ h\left(\left\{1 + \sum_{a\neq 0} \frac{w_k^{(a)} \hat{v}_a}{s}\right\}\right)\ \exp\left[-\beta \sum_{a=1}^{N} (J\lambda_a + g)\,\hat{v}_a^2\right] \end{aligned} \tag{S17}$$

where Z_s is the normalization of the distribution and the integral in $\mathrm{D}\hat{v}$ is over all the non-zero modes. We omitted all the irrelevant constants that cancel out through simplification between the distribution and its normalization. In the approximation where the relative fluctuations of the individual speeds are small, we can expand the function h appearing in the above expression and compute the remaining Gaussian integral for large values of N. We obtain, at leading order,

$$P(s) = \frac{1}{Z} s^{d-1} \exp\left[-\frac{Ng}{T}(s-v_0)^2\right] \ . \tag{S18}$$

We stress that the above approximation is quite reasonable in the deeply ordered phase. The quantity $\delta v_i = \sum_{a\neq 0} w_i^{(a)} \hat{v}_a$ indeed represents the fluctuation of the individual speed with respect to the mean speed of the group, s, and it must not be confused with the fluctuations of the mean speed itself. At low noise, when mutual adaptation is strong, individuals efficiently coordinate both their directions and speeds so that we expect individual deviations from the group mean flight direction (the polarization), and the mean speed to be small (as confirmed by simulations, see Fig. S1). On the other hand, if the value of g is small, i.e. the control on the individual speeds is loose, the $\{v_i\}$ can remain coordinated and at the same time wildly fluctuate (e.g. everyone speeds up), giving rise to large fluctuations of s, while keeping the relative deviations δv_i small.

The average value of the mean speed computed from distribution (S18) has been plotted in Fig.3b of the main paper: it predicts very nicely the values measured through numerical simulations of the off-lattice linear control model (see next section for details), confirming the validity of the approximations performed in the calculation (i.e. large directional order, quasi-equilibrium, small relative fluctuations of the speed). To get an analytical estimate of the typical speed, we can compute the maximum of the distribution. By imposing $\frac{\partial P}{\partial s} = 0$ for $d = 3$, we obtain the following equation,

$$s_{typical}^2 - s_{typical} v_0 - \frac{T}{Ng} = 0 \tag{S19}$$

that gives us the expression for the maximum:

$$s_{typical} = v_0 \left[\frac{1}{2} + \frac{1}{2}\sqrt{1 + \frac{4T}{Ngv_0^2}}\right] \ . \tag{S20}$$

This result confirms the idea that the mean speed is substantially different from v_0 for small N, if g is too small, as clearly shown in Fig.3b of the main paper.

We wish to draw the reader's attention on the fact that, despite the approximations we used to derive them (in particular the fixed network assumption), the analytical results of this section are in perfect agreement with numerical simulations performed by using an actual self-propelled particle model (see Fig.3b in the main text). This is not surprising, considering that in the deeply ordered flocking phase the time scale to reshuffle the interaction network is much larger than the time of local relaxation [1].

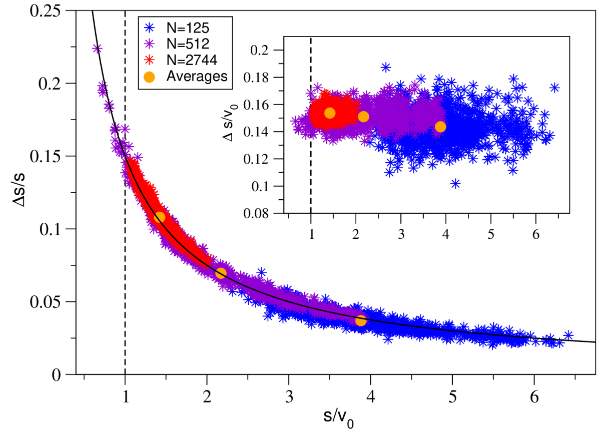

Fig. S1. Relative fluctuations of the speed. We report in this plot the relative fluctuations of the individual speed $\Delta s/s$ as a function of the mean speed, computed from numerical simulations for different values of N, and for $g = 10^{-3}$. The fluctuation is defined as $\Delta s = [(1/N)\sum_i \delta v_i^2]^{1/2}$. Each point in the plot corresponds to a distinct configuration, and all points of the same color are drawn from the same simulation performed at a given value of N. The big yellow points are averages over all data in the same simulation (i.e. N). The black line is a fit of the data with a $f(x) = a/x$ function. The vertical dashed line corresponds to $s = v_0$, which is the asymptotic value for the mean speed in the thermodynamic limit. The fluctuations themselves are small and depend on s only very weakly (inset), so that the *relative* fluctuations decay as $1/s$. Relative fluctuations therefore only increase due to the decrease of the average value of s at large sizes. However, such value is limited by below ($s < v_0$) and the relative fluctuations therefore remain small in the whole range of parameters.

Non-interacting and mean-field derivation

We analyze here two interesting limits of the linear speed control model, namely the non-interacting case and the mean-field case. The former gives an insight on the stochastic nature of the degree of freedom of the speed, while the latter provides a simpler derivation of the probability distribution studied in the previous section.
The non-interacting scenario is recovered when $J = 0$ and the equations of motion of the main text (1)(2) decouple in N independent equations. Writing explicitly the amplitude of the gaussian sources of noise, they appear,

$$\frac{d\mathbf{r}_i}{dt} = \mathbf{v}_i \tag{S21}$$

$$\frac{d\mathbf{v}_i}{dt} = -2g(|\mathbf{v}_i| - v_0)\frac{\mathbf{v}_i}{|\mathbf{v}_i|} + \sqrt{2T}\boldsymbol{\xi}_i \ , \tag{S22}$$

where now $\langle \boldsymbol{\xi}_i(t)\cdot\boldsymbol{\xi}_j(t')\rangle = d\delta_{ij}\delta(t-t')$. From these, we want to obtain a stochastic differential equation for the speed of the single particle, therefore we use spherical coordinates expressing, in $d = 3$, $\mathbf{v}_i(t) = s_i(\sin\theta_i\cos\varphi_i, \sin\theta_i\sin\varphi_i, \cos\theta_i)$ where θ_i and φ_i are the two phases of the vector. Within the Ito-scheme of stochastic calculus, we get the equation for the single the speed,

$$\frac{ds_i(t)}{dt} = -2g(s_i(t) - v_0) + \frac{2T}{s_i} + \sqrt{2T}\xi_i^s(t) \tag{S23}$$

where the noise term depends on the coordinates of the original noise and on the phases,

$$\xi_i^s = [\sin\theta_i(\cos\varphi_i\xi_i^x + \sin\varphi_i\xi_i^y) + \cos\theta_i\xi_i^z] \ , \tag{S24}$$

thus becoming a multiplicative noise with zero mean. This is a feature of the Ito approach, that also produces the additional drift term in the equation (S23) since, applying the rules of derivation, one obtains that,

$$\frac{ds_i(t)}{dt} = \frac{1}{s_i(t)}\left(\sum_\alpha^d v_i^\alpha(t)\frac{dv_i^\alpha(t)}{dt} + T(d-1)\right) \ . \tag{S25}$$

This picture tells us that, even if the original dynamics of the velocity contains the simplest gaussian noise, the dynamics of the speed is ruled by non-trivial equations and by multiplicative stochastic sources. The same computations can be performed using the marginal model deterministic force (Eq. (21) of Methods) and they give the same result: the effective noise acting on the speed degree of freedom is multiplicative. Averaging on the number of particles, we can compute the evolution of the mean speed $s = 1/N \sum s_i$, namely,

$$\frac{ds(t)}{dt} = -2g(s(t) - v_0) + (d-1)T(\overline{1/s}) + \sqrt{\frac{2T}{N}}\eta(t) \tag{S26}$$

where with $(\overline{1/s})$ we mean $1/N \sum_i (1/s_i)$, and η is again a white noise. Under the reasonable approximation that fluctuations around the mean value are small, that means we can write $s_i = s + \delta s_i$ with $\delta s_i / s_i \ll 1$, then we have,

$$\frac{1}{N}\sum_i \frac{1}{s_i} = \frac{1}{N}\sum_i \frac{1}{s}\left(1 - \frac{\delta s_i}{s}\right) = \frac{1}{s} \ ,$$

leading to a stationary probability distribution for the mean speed of N non-interacting particles equal to,

$$P(s) \sim s^{N(d-1)} e^{-\frac{gN}{T}(s-v_0)^2} \ , \tag{S27}$$

which seems quite similar to (S18), but with the difference that an N factor in the first contribution appears, thus preserving $s_{typical(i)} = s_{typical}$, if $s_{typical(i)}$ is computed with the single particle non-interacting distribution $P(s_i) \sim s_i^{(d-1)} e^{-\frac{g}{T}(s_i - v_0)^2}$. This means that, with this approximation, we cannot obtain the speed N-dependent result of eq. S20, which matches the SPP simulations result of Fig.3b of the main text.

On the other hand we can implement a slightly different approximation, which is more similar to the full spin-wave approach of the previous section, yet simpler. We claim that, due to the high polarization of the system, we can write every individual velocity as,

$$\boldsymbol{v}_i = \boldsymbol{v} + \boldsymbol{\epsilon}_i \ , \tag{S28}$$

where $\boldsymbol{v}$ is the average velocity $\boldsymbol{v} = 1/N \sum_i \boldsymbol{v}_i$, and $\boldsymbol{\epsilon}_i$ is the deviation of each individual velocity from the average, such that $|\boldsymbol{\epsilon}_i| \ll |\boldsymbol{v}|$. By definition we have $\sum_i \boldsymbol{\epsilon}_i = 0$. We plug (S28) into the Hamiltonian (S1), disregarding $\boldsymbol{\epsilon}_i$ (hence enforcing a mean-field velocity for every individual) and we obtain,

$$\mathcal{H}(\{\boldsymbol{v}_i\}) = g\sum_i (1-v)^2 = Ng(1-v)^2 \ . \tag{S29}$$

We stress the fact that also in this approximation the interaction term is neglected, but for the opposite reason with respect to the non-interacting case. Here we have only the potential because each vector $\boldsymbol{v}_i$ is very close to each vector $\boldsymbol{v}_j$, hence $(\boldsymbol{v}_i - \boldsymbol{v}_j)^2 \simeq 0$. In the non-interacting case we disregard the interaction, by enforcing $J = 0$, which eliminates the interaction term but implies that every vector $\boldsymbol{v}_i$ has a different orientation with respect to any vector $\boldsymbol{v}_j$. To compute the distribution of the collective variable $\boldsymbol{v}$, we need to change variable from $\{\boldsymbol{v}_i\}$ to $\{\{\boldsymbol{\epsilon}_i\}, \boldsymbol{v}\}$ (enforcing the constraint $\delta\left(\sum_i \boldsymbol{\epsilon}_i\right)$). This computation produces a constant that will cancel with the normalization of the probability distribution; in the end we have,

$$P(\{\boldsymbol{\epsilon}_i\}, \boldsymbol{v}) = \frac{e^{-\frac{Ng}{T}(1-v)^2}}{\int d\boldsymbol{v} \prod_i d\boldsymbol{\epsilon}_i \delta\left(\sum_i \boldsymbol{\epsilon}_i\right) e^{-\frac{Ng}{T}(1-v)^2}} \ . \tag{S30}$$

We integrate over the $\{\boldsymbol{\epsilon}_i\}$ and then we change variable from $\mathrm{d}\boldsymbol{v} = \mathrm{d}v_x \mathrm{d}v_y \mathrm{d}v_z$ to $\mathrm{d}v \mathrm{d}\phi \mathrm{d}\theta$, gaining the jacobian v^2. After integrating over the angular variables we have the probability distribution for v ($T = 1$),

$$P(v) = \frac{v^2 e^{-Ng(1-v)^2}}{\int dv \ v^2 \ e^{-Ng(1-v)^2}} \ , \tag{S31}$$

the variable v, if we use its definition from (S28), is $v = |\boldsymbol{v}_i - \boldsymbol{\epsilon}_i|$. Since we decided to completely ignore fluctuations, we conclude that $v = v_i$ and therefore $s = 1/N \sum_i v_i = v$. After the identification of s and v, the equation above coincides with the SW-approximation distribution (S18).

Distribution of the mean speed: marginal speed control

Let us now consider the marginal speed control model, that has the pseudo-Hamiltonian:

$$H(\{\boldsymbol{v}_i\}) = \frac{J}{2}\sum_{i,j} n_{ij}(\boldsymbol{v}_i - \boldsymbol{v}_j)^2 + \frac{\lambda}{v_0^6}\sum_i \left(v_i^2 - v_0^2\right)^4 \ . \tag{S32}$$

We can follow a similar procedure as the one used for linear control, i.e. we apply the SW approximation to deal with directional fluctuations and we decompose in normal modes for the speed fluctuations. We end up with a distribution with the same structure as the one of (S14),

$$H = \sum_{a=1}^{N} J\lambda_a \hat{v}_a^2 + \frac{\lambda}{v_0^6}\sum_i \left(\sum_{a,b} w_i^{(a)} w_i^{(b)} \hat{v}_a \hat{v}_b - v_0^2\right)^4 \ . \tag{S33}$$

Integration over the non-zero modes with this effective Hamiltonian is clearly a hard task, due to the non-Gaussian contributions. However, in the approximation where the relative speed fluctuations are small, things simplify: we can easily extract the zero mode contribution $\simeq N\frac{\lambda}{v_0^6}(s^2 - v_0^2)^4$ in the exponent, while at leading order the integration over the remaining modes (which is non-Gaussian in this case) will produce a constant integral. The distribution for the average speed s will then be:

$$P(s) = \frac{1}{Z} s^{d-1} \exp\left[-\frac{N\lambda}{Tv_0^6}(s^2 - v_0^2)^4\right] \ . \tag{S34}$$

The agreement between theory and simulations is less accurate than in the linear speed control case, but we still have a satisfying match between the predicted average mean speed and the value measured from numerical simulations (Fig.3d of the main paper). Once again we can compute the maximum of the distribution to estimate the typical mean speed for $d = 3$,

$$1 - \frac{4N\lambda v_0^2}{T}\left(\frac{s_{typical}}{v_o}\right)^2 \left(\left(\frac{s_{typical}}{v_0}\right)^2 - 1\right)^3 = 0 \ . \tag{S35}$$

Since we are interested in the behaviour of $s_{typical}$ in N at fixed T and λ, we can solve this equation in the two limits of big N and small N,

$$s_{typical} \simeq \begin{cases} v_0 \left[1 + \left(\frac{T}{32N\lambda v_0^2}\right)^{1/3}\right] & \text{for } N \gg \frac{T}{\lambda v_0^2} \\ v_0 \left(\frac{T}{4N\lambda v_0^2}\right)^{1/8} & \text{for } N \ll \frac{T}{\lambda v_0^2} \ . \end{cases} \tag{S36}$$

Polarization dependence on model parameters

In equilibrium ferromagnetic models in their low temperature phase, the polarization Φ only depends on the ratio between ferromagnetic coupling J and temperature T through the relation [2],

$$\Phi = 1 - \alpha\frac{T}{J} \tag{S37}$$

where α is a constant of order 1 whose value depends on the specific structure of the interaction network. This relation, though, is only valid when the vectorial degrees of freedom $\boldsymbol{v}_i$ have modulus 1, whereas if they have modulus equal to v_0 the relation changes to,

$$\Phi = 1 - \alpha\frac{T}{v_0^2 J} \ . \tag{S38}$$

For out-of-equilibrium models, as the present case, the polarization depends in principle on all parameters; however, in the deeply ordered phase that we are considering here, the main contribution to Φ is still given by the ratio between alignment and noise, so that (S38) remains a very useful rule of thumb to fix the parameters of the model such to have a polarization equal to that of natural flocks, namely $\Phi \simeq 0.89 \div 0.99$. To further justify the soundness of our approach we present another set of simulations, with different values of T and v_0^2, but with the ratio T/v_0^2 kept fixed, such that polarization (S38) remains untouched. These simulations are shown in Fig. S2, where we can see that the original simulations' phenomenology, reported in Fig. 3 of the main text, is reproduced.

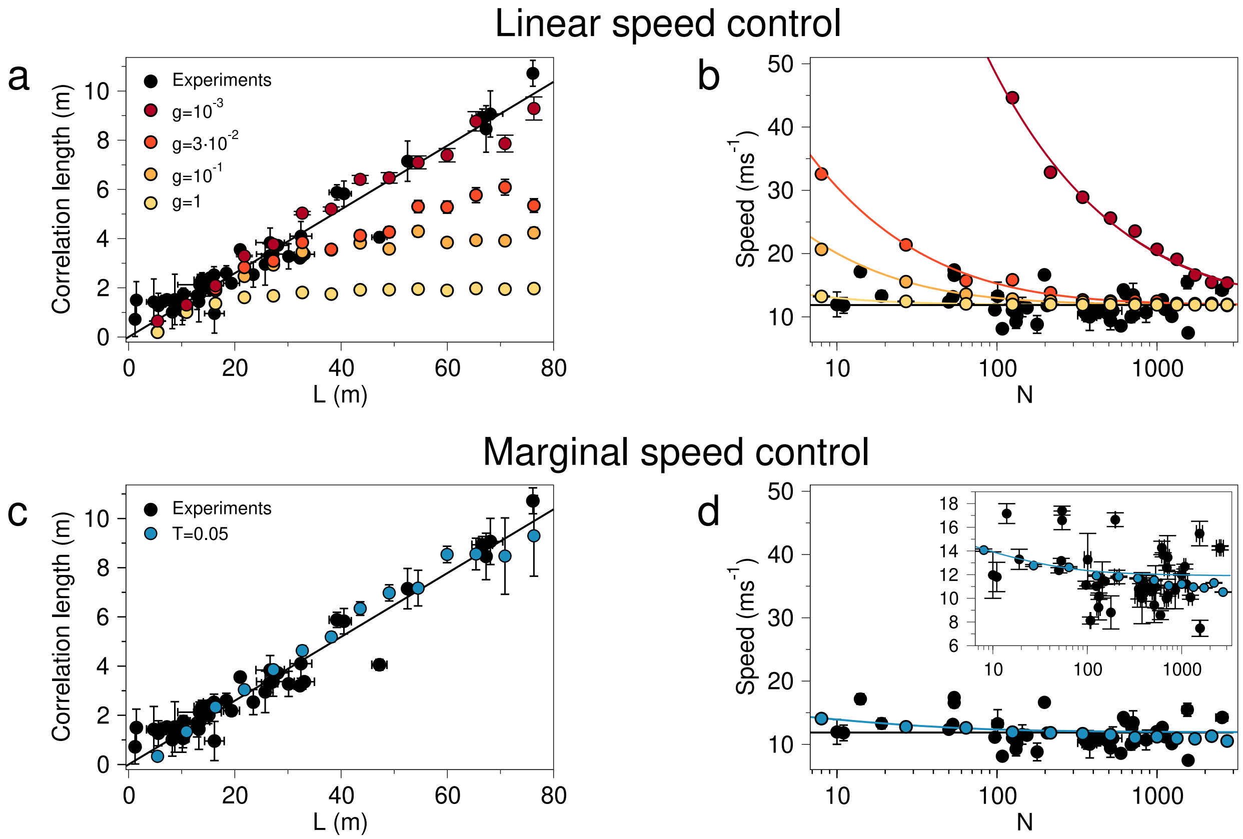

Fig. S2. Linear vs Marginal speed control - alternative set of parameters. The figure above shows the same results of Fig. 3 of the main text for a set of simulations where all the parameters were kept fixed except for the temperature $T' = T \times 400$ and the reference speed $v_0' = v_0 \times 20$, thus preserving the polarization (S38). The original values of T and v_0 are presented in Table S2.

Simulations with elongated geometry

As we pointed out in the main text, our simulations (Fig. 3 of the main text) are performed in a cubic volume with periodic boundary conditions. This choice was made in order to perform the most generic and simple type of simulation possible, without any biases that would have been introduced if we had chosen any particular non-symmetric shape. However, real flocks do not have a cubic aspect ratio [5], they are rather elongated in one direction. This poses the following problem: if a flock F and a simulation S have similar value of L (in units of interparticle distance), they will not have a similar value of N, because of the very elongated shape of the flock, compared to the cube; in fact, the cubic simulation will contain many more points than the elongated flock with the same linear extension L. Conversely, if F and S have similar N, they will have very different linear extensions L.

Hence, a possible objection to the way we present the data, in particular of the linear case, might be that our work is strongly influenced by the cubic shape in which we perform the simulations. The linear model run in a cubic simulation is shown to not fit the experimental data (Fig. 3a of the main text, light yellow points), but it is not trivial to predict how the linear model would behave in the more biologically plausible case of a non-cubic elongated geometry. To avoid any doubt we have run numerical simulations in non-cubic boxes, with elongated aspect ratio in the same range as natural flocks. In this new set of simulations, the volume in which the particles move is a box with a bigger edge L_1 and two smaller edges L_2, L_3 such that $L_2 = L_3$ and the aspect ratio L_1/L_2 spans from 6 to 8, mimicking flocks' aspect ratios. In this way, now if a flock F has linear extension L similar to that of simulation S, then also their number of particles N will be similar. We run these new simulations for the linear model and for the marginal model.

These new 'elongated' simulations (Fig. S3) show that the results do not change: the linear model cannot fit the experimental data, while the marginal model can. More precisely, in the linear model we fixed the stiffness at g=1, i.e. large enough to have the speed of the simulations under control (that is similar to v0) at all values of N, including

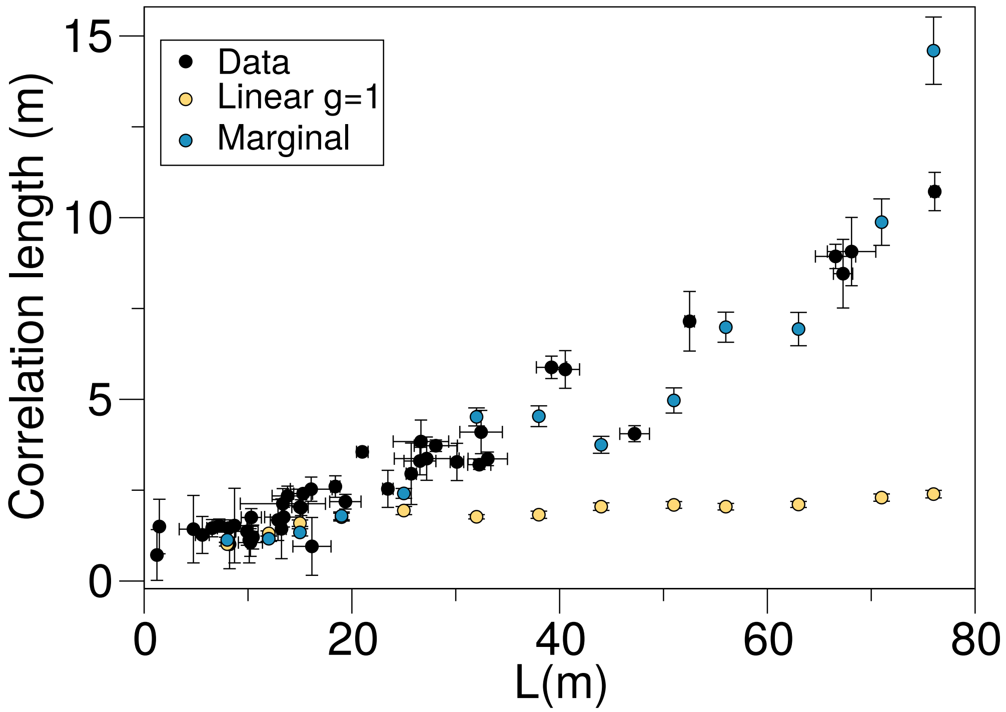

Fig. S3. Correlation length in simulations with elongated geometry. Linear and marginal simulations in an elongated volume, compared with experimental data. Correlation length against size of the system, computed as the distance between the furthest couple of particles in the system. All the simulations' parameters are the same as the ones reported in Tab. S2. These simulations are made in boxes with three axis L_1, L_2, L_3 such that $L_2 = L_3 < L_1$ with an aspect ratio L_1/L_2 spanning from 6 to 8, as in real flocks [5]. The result is the same as the original simulations in a cubic geometry: once we choose for the linear model a g big enough such that the speed is compatible with experiments, the correlation length does not scale with the system's size (yellow points). On the other hand the marginal model's speed correlation length (blu points) scales with the size of the system for all the sizes that we measure in experiments.

the lowest values of N (a limited mean speed of the group is really the biggest concern for the simulations to be realistic: the speed of the numerical flock cannot be 10 times as large as the reference speed v0). Then we measured the correlation length in the elongated numerical flocks at each value of L, and found that it is not scale-free, even in the elongated geometry (yellow points). Conversely, the marginal model works as well in the elongated geometry as in the cubic one, giving a scale-free correlation and a moderate speed at all values of L and N (blue points).

Is the marginal theory tuned at criticality?

An interesting question is whether or not a theory with marginal speed control requires any tuning of the parameters, and in particular tuning close to criticality [6]. The success of the marginal theory is based on the fact that the second derivative of the potential is exactly zero at v_0, a condition that seems to require some tuning. However, a small non-zero quadratic term would still be acceptable in the marginal case, as long as its amplitude is much smaller than $1/L^2_{\mathrm{max}}$; and thanks to the steep nonlinear rise of the marginal potential, there is no lower bound for it, so that this tuning is therefore rather lukewarm. On the other hand, the marginal theory requires the system to be close to the zero-temperature critical point, so in this sense there is a case for near-criticality. However, a zero-temperature critical point is not shifted by finite-size effects,it has just one physical side (positive temperature); hence, we can just push the system at low temperature, without worrying to *cross* the critical point in any way; as a result, the control parameter does not need to depend on size to keep the system close to criticality. As we have

seen, the situation would be different in the case of linear control, as the speed stiffness must be carefully tuned in a size-dependent way to remain close, but not too close, to the critical point.

Robustness against flocks' heterogeneities

When considering biological systems, statistical models like those we are using could appear oversimplified or lacking of one of the fundamental traits of living entities, namely heterogeneities in a group. Here we show the results of numerical simulations performed to test the robustness of the marginal model's predictions when some keystone individuals behave differently from the group.

We take under considerations two different types of realizing heterogeneity in our model:

i) birds may not all have the same reference speed v_0, but there can be individuals which travel faster (or slower) compared to the group, fluctuating as the others (i.e. λ fixed) around varied reference values, namely,

$$v_0^i = v_0(1 + \delta v_0^i) \tag{S39}$$

where i indicates only the outlier individuals, and δv_0^i the shift of the speed control potential's barycenter that is assumed equal for all the keystone birds. If δv_0^i is positive, this situation represents the presence of one or more leaders in the group, or else younger individuals that, when flying isolated, on average travel faster than the average starling. On the other hand, when δv_0^i is negative, it pictures the opposite scenario. We verified that simulations' results are independent on the sign of the perturbation δv_0^i, and therefore we are going to show what happens only for positive values of δv_0^i;

ii) birds can fluctuate around the same reference speed in different ways, due to some mechanical and biological dissimilarities. This scenario can be modeled by considering a perturbation in the amplitude of the speed potential, i.e.,

$$\lambda^i = \lambda(1 + \delta\lambda^i) \tag{S40}$$

while keeping fixed the reference speed v_0. We consider particles that can fluctuate more with respect to the mean behavior of the group, namely with $\delta\lambda^i$ less than zero and greater than -1.

We performed therefore sets of simulations for the two corresponding heterogeneities, allowing the presence of 1 and 3 keystone individuals in the group; the perturbation $\delta v_0^i = \delta v_0$ and $\delta\lambda^i = \delta\lambda$ are assumed equal for all the outliers.

Results are shown in terms of histograms of single individual speed distributions in Fig S4, where top panels regards the first method i), while bottom panels concern changing of speed variability, namely method ii). In panels **a** and **d**, we show as a reference the distributions of single isolated particles when the disturbance is applied, compared to the standard case where there is no variation. The effect to change the reference speed (panel **a**) is to accordingly increase (o decrease) the typical value of the single particle distribution, while its width is preserved. On the other hand, if we modify the parameter λ (panel **d**), what changes is not only the amplitude of speed fluctuations but also the mean value of the distribution. We chose $\delta v_0 = 0.3$, which corresponds to a 30% increase in the typical velocity of the keystone individuals, and $\delta\lambda = -0.99$, which corresponds to a reduction of 100 times of the control parameter. We opted for these values in order to simulate a relevant difference between the outliers' behavior and a standard individual, as it is evident from panels **a** and **d**. In the remaining panels we plot single particle distributions measured when one or three outliers of type i) (panel **b** and **c**) or ii) (panel **e** and **f**) are placed in a flock of standard individuals with reference speed $v_0 = 1$ and control parameter $\lambda = 10^{-3}$. The system evolves with high interaction amplitude $J = 10$ and $T = 0.5$, thus producing a coherent flock with polarization $\phi = 0.98$. Both in the cases of one single keystone individual and three of them, the speed distributions of the outliers and of a standard particle in the group are completely superimposable. These results therefore highlight that the dynamics expressed by the marginal model is able to resist to the introduction of heterogeneities in speed's variability and reference value, since outlier individuals, with different behaviors if picked isolated, are acted upon the group thus uniforming their motion to the collective one.

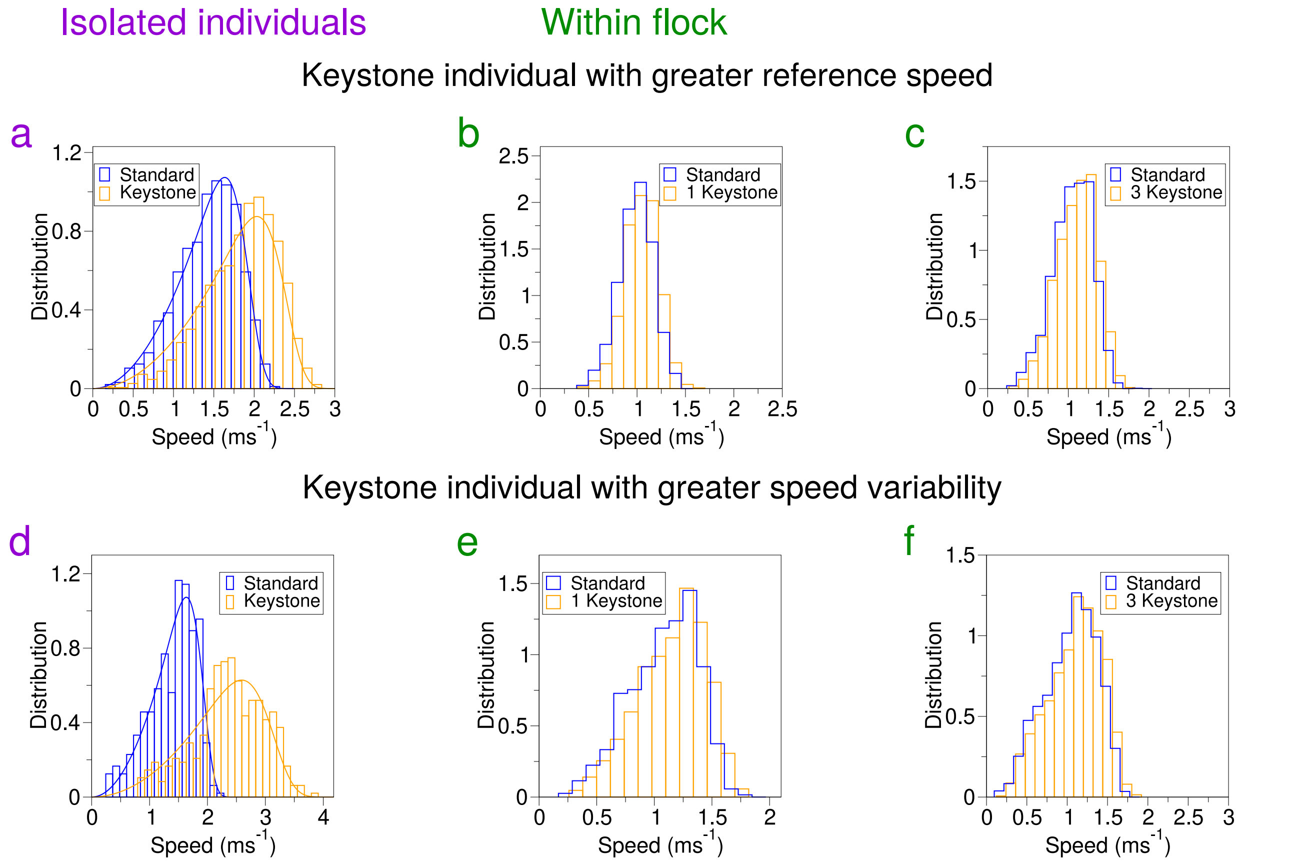

Fig. S4. Heterogeneities: single bird speed histograms for the marginal model. We show some speed histograms in various cases related to the introduction of heterogeneities in the flock. The histograms come from simulations while the solid smooth lines are simply the Boltzmann distributions $P \sim e^{-V/T}$, where V is the marginal potential of eq. 11 (main text). All these histograms are made from simulations of the marginal model with $T = 0.5$. Histograms in panel **a** and **d** come from simulations of isolated individual birds, while in panel **b**, **c**, **e** and **f** we show results from a simulated flock with $N = 125$ individuals. **a**: We compare an isolated standard individual speed distribution (blue) with an isolated keystone individual speed distribution (orange). The standard individual has typical speed $v_0 = 1$ and potential amplitude $\lambda = 10^{-3}$ while the keystone individual has enhanced reference speed $v_0 = 1.3$ and standard $\lambda = 10^{-3}$. We can see that the orange distribution is shifted to the right with respect to the blue one, meaning that the keystone individual moves faster than the standard one. **b**: We place the keystone individual of panel **a** inside a simulated flock of 124 standard individuals, with a nearest neighbors interaction strength $J = 10$. We can see that the speed distribution of the keystone individual, due to the interaction with the other birds in the flock, becomes identical (within fluctuations) to the standard individual speed distribution. **c**: Same procedure and same result of panel **b**, but with 3 keystone individuals in the flock instead of only 1. **d**: Same situation as panel **a**, but this time the keystone individual has $v_0 = 1$ and lowered potential amplitude $\lambda = 10^{-5}$. This produces enhanced speed fluctuations together with a larger average speed for the keystone individual. **e**: We place a keystone individual with enhanced speed variability (orange one of panel **d**) in a flock of standard individuals and we find once again that, due to the interaction, the speed distribution of a keystone individual is indistinguishable from the speed distribution of a standard bird. **f**: Same procedure as panel **e** but with 3 keystone individuals. The result stays the same: the keystone individuals, that have larger fluctuations and mean speed when they fly alone, are acted upon the group and their behavior is reduced to that of standard individuals. The marginal model is hence robust against flocks heterogeneities.

Acquisition	No. of birds N	Flock's size L, m	Polarization Φ	Mean speed s, m s^{-1}	Correlation length ξ, m
16-05	1548	68.1	0.961	15.5	9.1
17-06	380	40.5	0.935	10.0	5.8
21-06	530	26.6	0.973	11.0	3.8
25-08	1079	52.5	0.962	12.7	7.1
25-10	696	30.1	0.991	12.6	3.3
25-11	854	33.1	0.957	10.7	3.4
28-10	1122	32.3	0.982	11.2	3.2
29-03	422	28.1	0.963	10.8	3.7
31-01	1565	67.3	0.921	7.5	8.5
32-06	690	18.4	0.981	10.0	2.6
42-03	366	27.2	0.979	10.2	3.4
48-17	709	25.7	0.886	13.5	3.0
49-05	636	15.1	0.995	13.7	2.0
54-08	2548	66.6	0.971	14.2	8.9
57-03	2559	76.1	0.978	14.3	10.7
58-06	351	19.4	0.987	10.8	2.2
58-07	445	15.3	0.977	10.9	2.4
63-05	712	47.2	0.978	10.3	4.1
69-09	206	13.4	0.985	11.8	1.8
69-10	994	32.4	0.987	12.0	4.1
69-13	1238	39.2	0.937	10.1	5.9
69-19	617	21.0	0.975	14.3	3.6
72-02	101	7.2	0.993	13.3	1.5
77-07	131	6.5	0.978	9.2	1.5
20110208_ACQ3	178	12.9	0.983	8.8	1.7
20110211_ACQ1	595	23.5	0.971	8.6	2.6
20110217_ACQ2	405	15.0	0.982	11.1	2.0
20111124_ACQ1	125	8.1	0.993	11.0	1.5
20111125_ACQ1	50	8.7	0.983	12.4	2.0
20111125_ACQ2	512	26.6	0.956	9.4	3.3
20111201_ACQ3_F1	133	8.2	0.973	10.2	1.0
20111201_ACQ3_F4	488	16.2	0.972	10.6	1.0
20111207_ACQ1	108	13.8	0.931	8.1	2.3
20111214_ACQ4_F1	154	10.3	0.992	11.4	1.8
20111214_ACQ4_F2	144	13.4	0.968	11.6	2.2
20111215_ACQ1	391	16.1	0.984	11.1	2.5
20111220_ACQ2	198	10.2	0.985	16.6	1.2
20191209_ACQ53	97	9.9	0.991	11.1	1.4
20191209_ACQ55_F1	19	5.6	0.996	13.3	1.3
20191209_ACQ58_F2	53	10.5	0.988	13.2	1.3
20191209_ACQ58_F3	14	1.5	0.998	17.2	1.6
20200129_ACQ3	54	10.1	0.998	17.4	1.1
20200129_ACQ4_F1	11	4.7	0.988	11.8	1.5
20200129_ACQ4_F2	54	13.2	0.994	16.6	1.5
20200211_ACQ7	10	1.2	0.995	12.0	0.8

Table S1. Experimental data. This table reports all the data required to perform the analysis presented in this paper. Each line corresponds to a different acquisition (i.e. flocking recording), for all the three experimental campaigns considered (acquisitions labeling system changed from one campaign to another). Acquisitions belonging to different campaigns are separated by a straight line. For each acquisition we have: the median number of individuals N, the median flock's size L, the mean polarization $\Phi = 1/N\,|\sum_i \boldsymbol{v}_i/v_i|$, the median of the mean speed $s = 1/N \sum_i v_i$ and the median correlation length ξ, computed via (17) of the main text (Methods). Every median (or mean), relative to a particular acquisition, is made over all the frames in that recording. Since the measured polarization value depends on time resolution (higher resolution bringing more noise), acquisition of the second and third campaign (that are acquired at much faster rates) have been re-sampled at the same rate of the first campaign so as to have homogeneous measurements for all the data.

Speed control	g	λ	T	J
Linear	0.001	-	2.5×10^{-3}	10
	0.03	-	2.5×10^{-3}	10
	0.1	-	2.5×10^{-3}	10
	1.0	-	2.5×10^{-3}	10
Marginal	-	0.001	1.25×10^{-4}	1.0

Table S2. Parameters of simulations. In this table we report the values of relevant parameters used in the numerical simulations (results in Fig. 3 of the main text). The other parameters are $r_c = 1.2$, $v_0 = 0.05$, $\Delta t_{MRG} = 0.01$, $\Delta t_{GAUSS} = 0.001$.

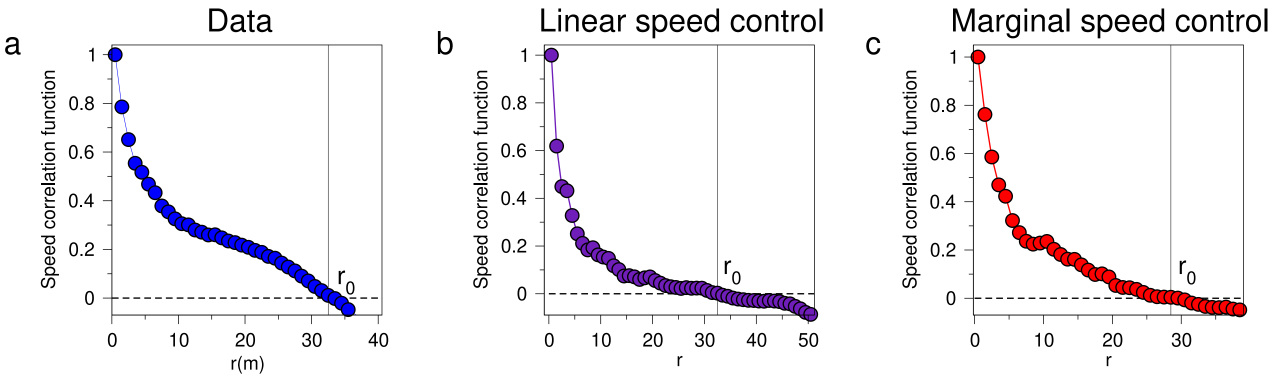

Fig. S5. Some examples of connected correlation functions. We report some examples of speed connected correlation functions, computed by (15) of the main text (Methods). The first point of zero-crossing (r_0) is visible for each function. All the functions are normalized such that $C(r = 0) = 1$. **a**: Example of speed connected correlation function in experimental data. **b**: Example of speed connected correlation function in linear speed control model simulations. **c**: Example of speed connected correlation function in marginal speed control model simulations. For **b** and **c** the distance r is measured in simulation units.

Supplementary References

[1] Mora, T. *et al.* Local equilibrium in bird flocks. *Nature Physics* **12**, 1153–1157 (2016).

[2] Dyson, F. General theory of spin-wave interactions. *Physical review* **102**, 1217 (1956).

[3] Bialek, W. *et al.* Statistical mechanics for natural flocks of birds. *Proc Natl Acad Sci USA* **109**, 4786–91 (2012).

[4] Bialek, W. *et al.* Social interactions dominate speed control in poising natural flocks near criticality. *Proceedings of the National Academy of Sciences* **111**, 7212–7217 (2014).

[5] Ballerini, M. *et al.* Empirical investigation of starling flocks: a benchmark study in collective animal behaviour. *Anim Behav* **76**, 201–215 (2008).

[6] Mora, T. & Bialek, W. Are biological systems poised at criticality? *J Stat Phys* **144**, 268–302 (2011).
