# Supplementary material for: Marginal speed confinement resolves the conflict between correlation and control in collective behaviour
Source: Nat Commun. 2022 May 10;13:2315. doi: 10.1038/s41467-022-29883-4 (PMC9090766; doi:10.1038/s41467-022-29883-4)
Supplement: Supplementary file 2 — Editorial Assessment Report [file 41467_2022_29883_MOESM2_ESM.pdf]

nature portfolio

GUIDED OPEN ACCESS

nature physics | nature communications | communications physics

Contents of this report

- **Manuscript details**: overview of your manuscript and the editorial team.
- **Review synthesis**: summary of the reviewer reports provided by the editors.
- **Editorial recommendation**: personalized evaluation and recommendation from all 3 journals.
- **Annotated reviewer comments**: the referee reports with comments from the editors.
- **Open research evaluation**: advice for adhering to best reproducibility practices.

About the editorial process

Because you selected the **Nature Portfolio Guided Open Access option**, your manuscript was assessed for suitability in three of our titles publishing high-quality work across the spectrum of physics research: *Nature Physics, Nature Communications* and *Communications Physics*. More information about Guided Open Access can be found here.

Collaborative editorial assessment

Your editorial team discussed the manuscript to determine its suitability for the Nature Portfolio Guided OA pilot. Our assessment of your manuscript takes into account several factors, including whether the work meets the **technical standard** of the Nature Portfolio and whether the findings are of **immediate significance** to the readership of at least one of the participating journals in the Nature Portfolio Guided Open Access physics cluster.

Peer review

Experts were asked to evaluate the following aspects of your manuscript:

- **Novelty** in comparison to prior publications;
- **Likely audience** of researchers in terms of broad fields of study and size;
- **Potential impact** of the study on the immediate or wider research field;
- **Evidence** for the claims and whether additional experiments or analyses could feasibly strengthen the evidence;
- **Methodological detail** and whether the manuscript is reproducible as written;
- Appropriateness of the literature review.

Editorial evaluation of reviews

Your editorial team discussed the potential suitability of your manuscript for each of the participating journals. They then discussed the revisions necessary in order for the work to be published, keeping each journal's specific editorial criteria in mind.

Journals in the Nature portfolio will support authors wishing to transfer their reviews and (where reviewers agree) the reviewers' identities to journals outside of Springer Nature.

If you have any questions about review portability, please contact our editorial office at guidedoa@nature.com.

nature portfolio

GUIDED OPEN ACCESS

nature physics | nature communications | communications physics

Manuscript details

Tracking number	Submission date	Decision date
GUIDEDOA-21-00102	20 April 2021	17 June 2021

**Title**	Marginal speed confinement resolves the conflict between correlation and control in natural flocks of birds	**Corresponding author**	Antonio Culla<br>**Affiliation:** Sapienza University of Rome
**Preprint information**	There is a preprint of this manuscript posted at https://arxiv.org/abs/2101.09748	**Peer review type**	Single-blind

Editorial assessment team

**Primary editor**	**Abigail Klopper**<br>**Journal:** *Nature Physics*, ORCID: 0000-0002-1571-2444<br>**Email:** a.klopper@nature.com
**Editorial team members**	**Arianna Bottinelli**, *Communications Physics*, ORCID: 0000-0002-0460-7673<br>**Iryna Omelchenko**, *Nature Communications*, ORCID: 0000-0002-0449-6213
**About your primary editor**	Abigail joined *Nature Physics* in 2011, following a postdoctoral career at the Max Planck Institute for the Physics of Complex Systems in Dresden, Germany, in which she pursued theoretical research in aspects of soft-matter and biological physics. She obtained her PhD from the University of Western Australia in 2006, investigating dimensional effects in frustrated magnetic materials. Abigail is based in the London office.

Editorial assessment and review synthesis

**Editors' summary and assessment**	Most theoretical models predicting that collective animal behaviour can emerge through local interactions implement a linear control mechanism to keep the speed of individuals close to some biologically plausible value. Here, the authors use empirical data and an adaptation of the Vicsek model to show that linear speed control is not compatible with the long-range correlations in speed fluctuations observed in real starling flocks. Instead, they suggest that a nonlinear speed control mechanism they proposed in an earlier paper offers a better description of the data. They discuss the plausibility of their theory in the context of avian behaviour.
**Editorial synthesis of reviews**	Referee 1 expresses concerns about the degree of the advance with respect to previous work, and the clarity of presentation. Referee 2 questions the biological relevance of the findings, as well as the model assumptions, and suggests that the theory be directly compared to the data. The referee also notes omissions in the references, and suggests a simpler route to obtaining the authors' derivation. Referee 3 echoes the concerns regarding biological relevance, and offers guidelines for expanding the discussion.<br><br>The concerns raised by referee 2 regarding the assumptions of the model are crucial for publication in any Nature Portfolio journal. In addition to addressing the concerns of this referee, if the authors improve the clarity of presentation in response to referee 1, and make clear which assumptions are based on biological evidence, the paper would be suitable for *Communications Physics*. If they are also able to comment more extensively on the biological relevance along the lines provided by referee 3, and demonstrate the robustness of their model with respect to flock heterogeneities, the paper would be suitable for *Nature Communications*.<br><br>The authors should edit the text to be more transparent about the relevance of ref. 35, and respond to the queries from referee 1 regarding their figures, parameter choices, and their justification for the failure of linear control. A discussion of the model's limitations would be welcome, as would a direct comparison of the theory with data. A simplification of the derivation should also be considered by the authors.

Editorial recommendation

nature physics	Revision not invited	This study may well attract interest within the specialist community working on models for flocking, but the appeal is limited outside that group, particularly in light of the authors' previous publication detailing the model.
nature communications	Major revisions	The work may be suitable for this journal if the authors can respond comprehensively to the points raised in all three reports.
communications physics	Minor revisions	The work may be suitable for this journal if the authors can respond to the concerns of referees 1 and 2, and comment briefly on the issue of biological relevance raised by referee 3.

Next steps

Recommendation summary

- **Option 1:** Revise for *Nature Communications* by responding to the comments made by all three referees, paying particular attention to the concerns of referee 2 regarding the assumptions and limitations of the study.
- **Option 2:** Revise for *Communications Physics* by responding to the comments made by referees 1 and 2, and commenting briefly on the issue of biological relevance raised by referee 3.

See the previous page for details

Revision

If you would like to follow our recommendation, please upload the revised manuscript, along with your point-by-point response to the reviewers' reports and editorial advice **using this link***.

*This URL links to your confidential home page and associated information about manuscripts you may have submitted, or that you are reviewing for us.

Revision checklist

- Cover letter, stating to which journal you are submitting
- Revised manuscript
- Point-by-point response to reviews
- Updated reporting summary and editorial policy checklist
- Supplementary materials (if applicable)

Submission elsewhere

We can share the reviews with another journal outside of the Nature Portfolio if requested. You will need to request that the receiving journal office contacts us at guidedoa@nature.com. We have included editorial guidance below in the reviewer reports and open research evaluation to aid in revising the manuscript for publication elsewhere.

Annotated reviewer reports

The editors have included some additional comments on specific points raised by the reviewers below, to clarify requirements for publication in the recommended journals. However, please note that all points should be addressed in a revision, even if an editor has not specifically commented on them.

Reviewer #1	
**Reviewer #1**	This reviewer has chosen to remain anonymous. The reviewer's identity can only be shared with representatives of an established journal editorial office.
**Reviewer #1 expertise**<br>Summarised by the editor	Collective behaviour theory
**Editor's comments about this review**	This referee expresses concerns about the degree of the advance with respect to previous work, and the clarity of presentation.
**Reviewer #1 comments**	
**Overview**	The title of the manuscript does provide a good summary of the work: "Marginal speed confinement resolves the conflict between correlation and control in natural flocks of birds". The authors first explain what they mean by the 'conflict' between correlations and speed control. They then proceed to argue that the linear speed control often used in models of animal groups cannot be faithful to their data gathered on real bird flocks. They then proposed a different, 'marginal' speed control mechanism, which they argue does account for the data.<br>The idea is not new. A subset of the current authors published it in Comptes Rendus Physique (ref[35] in the present manuscript), but the reader does not learn about this before the end of page 5, i.e. quite late in the main text. As mentioned by the authors, ref[35] does not attempt to apply the idea and calculations made there to real flocks, nor does it present particle-level simulations, but the motivation and the main idea are presented there.<br>Beyond this, the paper is reasonably well presented, the problem is well explained, and the results could be of interest beyond the immediate subcommunity of people working on collective motion of real animal groups.

Specific comments		
**#**	**Reviewer comment**	**Editorial comment**
**1**	Impact<br><br>Given the above, I do not see this paper in Nature Physics. It might be suitable for Nature Communications, especially since this journal covers all of science, not just physics, something valuable in the present case.<br><br>The paper (but also its 'predecessor' ref[35]) might influence theorists working in active matter.	
**2**	Strength of claims<br><br>Even though the paper is well presented and well written, it suffers, in my opinion, of several problems in its presentation: a lot of the paper is about simulations of the particle-based model presented, and more precisely about simulations tailored to convince the reader that the model accounts well for data taken on a large ensemble of bird flocks of different size. This 'tailoring' (by which I mean how did the authors choose the parameters they use for each real flock) is relegated to the SI, in a rather intricate paragraph entitled "Gauging the values of N and L in numerical simulations". Given the central importance of these choices to support the conclusions put forward by the authors, I find this unacceptable practice. The SI could have contained data showing what happens when one makes other choices, but that is not available.	
**3**	I find that Figure 1 could carry much more (and more interesting) information than in its current form. For instance, scatter plots of speed vs polarization, speed vs group size, polarization vs group size	
**4**	In Fig.1b (and also in Fig.2) I find the symbol size too large, 'hiding' information	
**5**	Fig.2d is quite ridiculous as it stands, with its inset showing better the exact same data as its main panel, whose scales seem chosen to lead the reader into believing that indeed speed is independent of group size.	

6	Do the data in Fig.2a indicate that the speed correlation length is only about one tenth of the size of the flock? If this is correct, can the authors comment on this?	
7	The important choices made when selecting Eq(3) are not discussed. It is annoying that Eq.(3) be presented as some obvious unique choice. Eq.(3) implies that speed and orientation are not explicitly coordinated. How about discussing models where they would be?	
8	Somewhat similarly, the results about the 'failure' of linear speed control, in particular Eq.(6) and (7) could/should be discussed. E.g. can't one imagine that flocks 'regulate' their speed control so as to keep g*N constant? Would be 'work'?	
9	Overall, I suggest the authors tone down their claims that the marginal control mechanism they propose is "the right way" of accounting for their data. It may be one way of doing this. I believe others can be imagined. It would actually be nicer if the authors could comment on this at the end of the paper.	
10	<u>Reproducibility</u><br>Overall, it should be possible for someone to reproduce the numerical results (but the actual info to do this is buried in the SI). Regarding the analysis of the real flocks, the authors have explained before what exactly they are doing to calculate correlations, etc.	

Reviewer #2	
**Reviewer #2**	This reviewer has chosen to remain anonymous. The reviewer's identity can only be shared with representatives of an established journal editorial office.
**Reviewer #2 expertise**<br>Summarised by the editor	Collective behaviour theory, collective information processing
**Editor's comments about this review**	This referee questions the biological relevance of the findings, as well as the model assumptions, and suggests that the theory be directly compared to the data. The referee also notes omissions in the references, and suggests a simpler route to obtaining the authors' derivation.

Reviewer #2 comments	
**Overview**	In the submitted manuscript the authors study the flocking dynamics of starling flocks and the conflict between scale-free correlations in speed fluctuations and control of the mean speed to a reference value.<br>Overall the manuscript is very well written. It appears methodologically well done, as to be expected from such an expert group of authors on the topic of statistical physics applied to flocking. Also the data set on starling flocks is clearly unique, and the authors also added some recent data sets expanding the size range of the flocks. Overall the paper makes, for the model(s) considered, convincing arguments regarding the issues with linear speed control, as well as, how assuming a marginal speed control appears to resolve the problems. The figures are well done and help the comprehension of the manuscript.<br>However, there are some conceptual issues, e.g. related to the fundamental assumptions of the model(s) (see details below), which potentially could point towards alternative explanations for the empirical data. Thus the very strong conclusions towards the marginal speed control may not be warranted. At least these conceptual issues/limitations must be addressed/discussed.<br>In general, the topic of the manuscript and the research question are certainly of interest to experts in the field of collective behavior, in particular those with a strong statistical physics background. However the broader relevance of these results, particularly important for an interdisciplinary high impact journal as Nature Communications, is not clear. The authors include a section on biological relevance, however appears not to discuss that much of biological relevance, but rather to provide a justification why potential energetic costs of marginal control may not be important for starlings. So it's more about plausibility than relevance. What is

completely missing is laying out why biologists or engineers should care whether it's a linear or marginal speed control in flocks. What are the consequences for self-organized collective behavior apart from reconciling certain observations in bird flocks, which maybe could be also reconciled through other mechanisms (see below). It is also not clear how relevant this may be to other flocking or schooling phenomena, if one moves beyond starling flocks as a model species. Overall, in the current form the manuscript, in the way it's written, appears to address a more specialized audience in statistical physics of biological systems, and thus may be more suited for a more specialized journal.

Specific comments

#	Reviewer comment	Editorial comment
1	Impact<br>There are no real arguments given why researchers, in particular in interdisciplinary context, should care about this difference between linear and marginal speed control apart from the reported discrepancy is resolved by marginal control. I am afraid this makes it easy for researchers to dismiss the potentially broader implications of this result, and consider this as a potentially isolated observation in starlings.	
2	Strength of claims<br>The empirical results suggest that the simple model with linear speed control, constant reference value v0, and vectorial, Gaussian white noise is not able to reproduce experimental observations. Thus clearly the model needs to be modified or extended. In this work the authors claim already in the abstract that<br>"the marginal speed confinement (...) is the only mechanisms reconciling scale-free correlations with biologically acceptable flock speed"<br>This is a very strong statement - and as such appears unjustified - as it can be easily questioned for the following reasons:<br>The authors assume a simple model in the spirit of statistical physics with additive, Gaussian white noise. However, birds are not spins with thermal noise, i.e. the fluctuations in real bird flocks are most surely neither	The concerns about the assumptions underlying your analysis should be clarified.

	completely additive, nor delta correlated in time and across individuals. For example, multiplicative noise may qualitatively change the behavior of the entire system including the behavior of the correlation length, dependence of mean speed on system size etc. Similar argument can be made if the fluctuation have some finite temporal correlations potentially depending in a non-trivial way on model parameters, or when the fluctuations between neighbors are correlated due to common environmental impacts (gusts of wind, visual or acoustic cues etc).	
3	Furthermore, the authors assume vectorial noise where a single noise strength parameter controls the noise in x and y independent on the birds direction of motion. Thus fluctuation in speed are always coupled to fluctuations in orientation. For birds it appears reasonable to assume that at least to a certain degree they are capable of adjusting their speed independent of their heading and vice versa, thus it is reasonable to assume that the same holds for corresponding fluctuations. More biologically inspired models of flocking and schooling distinguish this different types of noise (see e.g. Huth and Wissel, Journal of Theoretical Biology 3 (1992) https://doi.org/10.1016/S0022-5193(05)80681-2). At least one paper in physics seems to also makes this distinction (Grossmann et al, New Journal of Physics 14, 2012, https://doi.org/10.1088/1367-2630/14/7/073033). It appears that such noise in Cartesian formulation actually indeed leads to multiplicative noise terms.<br><br>The authors assume that v0 itself is a constant, but as we are dealing with biological agents, v0 itself could be a dynamical variable. I see a priori no reason why such a higher-order model should not in principle be able to reconcile the two allegedly conflicting observations. Thus the conclusion that marginal speed control is the ONLY explanation is not warranted, here definitely the authors should provide a more critical discussion of the potential limitations of their approach.	The limitations of your model and its potential extensions should be discussed.
4	There is also an obvious question regarding the methodological approach used in the paper: Given the model used, it should be possible to obtain analytically (with approximations), or at least via simulations the	Please consider extending the analytical model, or comparing the experimental distribution of

	actual distribution of individual speeds (not the average speed) for the linear and the marginal speed control, and compare them directly to the experimentally observed ones. If indeed only with the marginal control a good agreement is observed, this would be a much more direct and convincing results, potentially also refuting some of the points I made above.	individual speeds with simulations.
5	The authors write on line 147: "simplest control indeed the one used in virtually all models of fluctuating speed to date ... consists of a Gaussian potential confining the speed". First, I would call this a parabolic or harmonic potential and not a Gaussian one. Second, this statement ignores broader literature on self-propelled particles with variable speed, where often a cubic speed control is used, see e.g.:<br>- Niwa "Self-organizing dynamic model of fish schooling." Journal of theoretical Biology 171.2 (1994): 123-136.<br>- Erdmann et al. "Brownian particles far from equilibrium." The European Physical Journal B-Condensed Matter and Complex Systems 15.1 (2000): 105-113.<br>- D'Orsogna et al. "Self-propelled particles with soft-core interactions: patterns, stability, and collapse." Physical review letters 96.10 (2006): 104302.<br>- Hanke et al "Understanding collective dynamics of soft active colloids by binary scattering." Physical Review E 88.5 (2013): 052309.	Please extend the discussion on the relationship between your results and recent findings on self-propelled particles with variable speed.
6	This leads me directly to the next point. In the consideration of different confining potentials for speed the authors only allow/consider potential symmetric with respect to v0. It appears to me that for birds, or animals in general accelerating should be harder than slowing down, thus in general corresponding speed potential could also be asymmetric. There appears no justification or discussion of this constraint.	Please discuss the impact that asymmetry would have if included in your model.
7	Finally, the results for the average speed distributions are pretty simple. Whereas in the SI a rather involved derivation is given, it appears the same results can be obtained rather easily by simply making a number of reasonable assumptions. The results essentially appear to	Please consider the suggested simplifications in the derivations.

	correspond to the distribution of the average speed of N particles with vanishing alignment interactions and with negligible angular fluctuations, which is well fulfilled in the highly aligned state. Then only speed control plays a role, and the distribution will be determined by the exponential of the speed potential divided by the intensity of the effective noise on the mean speed which can be simply assumed to be T/N. I highly value the proper derivation in the SI, but given the simplicity of the result one should point at least out that the same result can be obtained much easier.	
**8**	Reproducibility<br><br>The overall quality of the analysis of experimental and simulation data is good. However, researchers won't be able to reproduce these results without access to the experimental data	

Reviewer #3

**Reviewer #3**	This reviewer has chosen to remain anonymous. The reviewer's identity can only be shared with representatives of an established journal editorial office.
**Reviewer #3 expertise**<br>Summarised by the editor	Avian flocking behaviour, ecology
**Editor's comments about this review**	This referee expresses concerns about the biological significance of the work, and offers guidelines for expanding the discussion.

Reviewer #3 comments

**Overview**	This is a very interesting study. I commend the authors for the volume and quality of the data they have collected. Please note I'm only commenting on the biological side of the work as the modelling aspect is beyond the scope of my expertise. speed consensus within flocking in swarming animals has indeed been a topic that has been largely overlooked. In more recent years it has, however, garnered more attention time has previously been the case. Interestingly some of the recent empirical work has found at times conflicting results when factoring speed into collective behaviour. Some empirical studies have been replicated and validated by models while others have proved more elusive. As such this paper is very timely. I do believe it substantially adds to our current knowledge about this important parameter when considering collective behaviour.

Specific comments

#	Reviewer comment	Editorial comment
**1**	I would encourage the authors to consider increasing the section where they talk about the biological significance. This currently reads a little weak and perhaps almost like an afterthought. For example, the comment regarding high costs and the high associated increases in energy expenditure amongst our murmurating starlings prior to roosting is largely an assumption. To the best of my knowledge direct energetic costs of murmurating has not been directly measured in free flying wild animals. By	The biological relevance of your model should be justified, and its robustness with respect to homogeneities should be demonstrated.

	expanding this section it will make this paper more accessible and of more interest to a far wider audience. The main core finding that small variations in speed are essentially tolerated and considered not a big deal within a Starling flock is itself novel and interesting. Furthermore, that large deviations in speed are sharply acted upon is also interesting, although the latter finding is perhaps less surprising. An expansion of this section regarding biological significance of these findings would benefit by proposing what the next step should be beyond working for example with just Starling flocks. What would happen and flocks of birds, for example, where there is greater variation in structural size and body mass between individuals within the same species? Are higher speeds, or rather higher speed deviations, accepted and tolerated more in flocks comprising young birds? How context dependent do you believe your findings are? Do your findings apply only to murmurating styles? How widespread do you believe this phenomenon is? What would you predict the situation to be in mixed species flocks? What may happen if keystone individuals within the collective begin to deviate greater in their speed than other individuals? Will then the collective change to fit these keystone individuals, or will these keystone individuals similarly have their large speed deviation acted upon and reduced? What happens over longer duration flight and longer distances?	
**2**	I most definitely see the value and potential in this study. I do believe the biological aspects of it need working on. The sections read a little informal and lacks depth. I do appreciate that such broad scale modelling approaches inevitably result in broad scale findings. But it is tricky to ignore the substantial and ever-increasing literature on how certain individuals play a far greater role in a moving collective than others. I am by no means suggesting that your analysis should change in any way. I do believe the final section on biological significance would benefit from highlighting this aspect further.	

Open Research Evaluation

Data availability

Data availability statement

Please add a data availability statement including accession details for deposited data, directions for obtaining source data, and a statement that all other data are available from the corresponding author (or other sources, as applicable) on reasonable request. Please refer to our data availability policy and our guidance on formatting your statement for more information.

Source data

All source data underlying the graphs and charts presented in the main figures must be made available as supplementary data (in Excel or text format) or via a repository such as figshare or Dryad. This is mandatory for publication in a Nature Portfolio journal, but is also best practice for publication in any journal. In the present paper, Figures 1 and 2 should be accompanied by the underlying source data.

Data citation

Please cite any datasets stored in external repositories that are mentioned within their manuscript within the main reference list. For previously published datasets, we ask that you cite both the related research article(s) and the datasets themselves. For more information on how to cite datasets in submitted manuscripts, please see our data availability statements and data citations policy.

Citing and referencing data in publications supports reproducible research, by increasing the transparency and provenance tracking of data generated or analysed during research. Citing data formally in reference lists also helps facilitate the tracking of data reuse and may help assign credit for individuals' contributions to research. A number of Springer Nature imprints are signatories of the Joint Declaration on Data Citation Principles, which stresses the importance of data resources in scientific communication.

Code availability and citation

Please include a code availability statement indicating how the custom code/software reported in your study can be accessed, including any restrictions to access. This section should also include information on the versions of any software used, if relevant, and any specific variables or parameters used to generate, test, or process the current dataset. Code availability statements should be provided as a separate section after the data availability section.

Ideally, code should be deposited in a repository such as Zenodo, Gigantum or Code Ocean and cited in the reference list following the guidelines described in our policy pages (see link below). Authors are encouraged to manage subsequent code versions and to use a license approved by the open source initiative.

Please read our code availability policies for more information. You may also find our code and software submission checklist useful. Please note that this form must be opened and completed in Adobe Reader.

Ethics

Please provide a competing interests statement that either declares the competing interests of the authors, or states that there is none. See our competing interests policy for further information.

Statistics

Error bars should be displayed wherever possible and must be clearly defined in the caption for each figure.

Methods

The methods section should be written as concisely as possible, but should contain the information necessary to allow interpretation and reproduction of the results. We encourage you to include all key methods in the main manuscript, rather than in the Supplementary Information. This section can be up to 3,000 words in length, can contain references that do not count towards the reference limit in the main paper, and will be fully indexed. Please note that figures or tables pertaining to the methods section should be included as extended data. If there are additional references in the methods section, their numbering should continue from the last reference in the main paper.
