# Supplementary material for: Marginal speed confinement resolves the conflict between correlation and control in collective behaviour
Source: Nat Commun. 2022 May 10;13:2315. doi: 10.1038/s41467-022-29883-4 (PMC9090766; doi:10.1038/s41467-022-29883-4)
Supplement: Supplementary file 3 — Reporting Summary [file 41467_2022_29883_MOESM3_ESM.pdf]

nature portfolio

Corresponding author(s): Antonio Culla

Last updated by author(s): Feb 25, 2022

Reporting Summary

Nature Portfolio wishes to improve the reproducibility of the work that we publish. This form provides structure for consistency and transparency in reporting. For further information on Nature Portfolio policies, see our Editorial Policies and the Editorial Policy Checklist.

Statistics

For all statistical analyses, confirm that the following items are present in the figure legend, table legend, main text, or Methods section.

n/a	Confirmed	
☐	☒	The exact sample size (*n*) for each experimental group/condition, given as a discrete number and unit of measurement
☐	☒	A statement on whether measurements were taken from distinct samples or whether the same sample was measured repeatedly
☐	☒	The statistical test(s) used AND whether they are one- or two-sided<br>*Only common tests should be described solely by name; describe more complex techniques in the Methods section.*
☒	☐	A description of all covariates tested
☒	☐	A description of any assumptions or corrections, such as tests of normality and adjustment for multiple comparisons
☐	☒	A full description of the statistical parameters including central tendency (e.g. means) or other basic estimates (e.g. regression coefficient) AND variation (e.g. standard deviation) or associated estimates of uncertainty (e.g. confidence intervals)
☐	☒	For null hypothesis testing, the test statistic (e.g. *F*, *t*, *r*) with confidence intervals, effect sizes, degrees of freedom and *P* value noted<br>*Give P values as exact values whenever suitable.*
☒	☐	For Bayesian analysis, information on the choice of priors and Markov chain Monte Carlo settings
☒	☐	For hierarchical and complex designs, identification of the appropriate level for tests and full reporting of outcomes
☒	☐	Estimates of effect sizes (e.g. Cohen's *d*, Pearson's *r*), indicating how they were calculated

Our web collection on statistics for biologists contains articles on many of the points above.

Software and code

Policy information about availability of computer code

Data collection	To collect the data was used IDT proprietary software Motion Studiox64 (version 2.09.06 for the data collected in 2011-2012 and version 2.16.02 for the data collected in 2019-2020). No software was used for the data collected in 2007-2008. All procedures and algorithms that we used are not central to the research, therefore they are not available.
Data analysis	Data analysis and simulations were performed using custom code programs, written in C, C++ and bash. Also the Open Source Library opencv (version 4) and the IBM proprietary software package IBM ILOG CPLEX Optimization Studio were used in the analysis. Some theoretical computations were made using Mathematica. All procedures and algorithms that we used are not central to the research, therefore they are not available.

For manuscripts utilizing custom algorithms or software that are central to the research but not yet described in published literature, software must be made available to editors and reviewers. We strongly encourage code deposition in a community repository (e.g. GitHub). See the Nature Portfolio guidelines for submitting code & software for further information.

Data

Policy information about availability of data

All manuscripts must include a data availability statement. This statement should provide the following information, where applicable:

- Accession codes, unique identifiers, or web links for publicly available datasets
- A description of any restrictions on data availability
- For clinical datasets or third party data, please ensure that the statement adheres to our policy

The flocks data that were analysed in this study are provided in the Supplementary Information file (Table S1).

Field-specific reporting

Please select the one below that is the best fit for your research. If you are not sure, read the appropriate sections before making your selection.

☒ Life sciences ☐ Behavioural & social sciences ☐ Ecological, evolutionary & environmental sciences

For a reference copy of the document with all sections, see nature.com/documents/nr-reporting-summary-flat.pdf

Life sciences study design

All studies must disclose on these points even when the disclosure is negative.

Sample size	After the tracking procedure was concluded, the number of individuals in each flock was determined by averaging the number of individuals in each frame of the same flock recording.
Data exclusions	No data were excluded.
Replication	The experiment is completely reproducible as shown by data presented in the paper, which show a high compatibility even though collected in different years, from different researchers and with different hardware. We therefore affirm that all the attempts at replication were successful.
Randomization	We did not allocate participants into groups. The participation was random, meaning that we collected data on flocks in their natural environment without any direct control on the groups under investigation.
Blinding	Investigators were blinded to group allocation, because there was not group allocation during data collection and analysis.

Reporting for specific materials, systems and methods

We require information from authors about some types of materials, experimental systems and methods used in many studies. Here, indicate whether each material, system or method listed is relevant to your study. If you are not sure if a list item applies to your research, read the appropriate section before selecting a response.

Materials & experimental systems

n/a	Involved in the study	
☒	☐	Antibodies
☒	☐	Eukaryotic cell lines
☒	☐	Palaeontology and archaeology
☐	☒	Animals and other organisms
☒	☐	Human research participants
☒	☐	Clinical data
☒	☐	Dual use research of concern

Methods

n/a	Involved in the study	
☒	☐	ChIP-seq
☒	☐	Flow cytometry
☒	☐	MRI-based neuroimaging

Animals and other organisms

Policy information about studies involving animals; ARRIVE guidelines recommended for reporting animal research

Laboratory animals	The study did not involve laboratory animals.
Wild animals	The study is based on observations of wild starlings (sturnus vulgaris) flocks. The experimental technique used is stereoscopic photography, where multiple synchronized video-sequences of a flocking event are acquired from different observation points (at a distance of approximately 100m from the flocks) with a calibrated multi-camera video-acquisition system.
Field-collected samples	The study did not involve samples collected from the field.
Ethics oversight	No ethical approval or guidance was required, ther was not any direct interaction with the animals.

Note that full information on the approval of the study protocol must also be provided in the manuscript.
